# The Effect of Gentamicin-Induced Readthrough on a Novel Premature Termination Codon of CD18 Leukocyte Adhesion Deficiency Patients

**DOI:** 10.1371/journal.pone.0013659

**Published:** 2010-11-16

**Authors:** Amos J. Simon, Atar Lev, Baruch Wolach, Ronit Gavrieli, Ninette Amariglio, Ester Rosenthal, Ephraim Gazit, Eran Eyal, Gideon Rechavi, Raz Somech

**Affiliations:** 1 Cancer Research Center, Chaim Sheba Medical Center, Tel Hashomer, Israel; 2 Sackler Faculty of Medicine, Tel Aviv University, Tel Aviv, Israel; 3 Hematology Laboratory, Chaim Sheba Medical Center, Tel Hashomer, Israel; 4 Laboratory for Leukocyte Function and Department of Pediatrics, Meir Medical Center, Kfar Saba, Israel; 5 Tissue Typing Laboratory, Chaim Sheba Medical Center, Tel Hashomer, Israel; 6 Pediatric Immunology Service, Chaim Sheba Medical Center, Edmond and Lily Safra Children's Hospital, Tel Hashomer, Israel; Universidade de Sao Paulo, Brazil

## Abstract

**Background:**

Leukocyte adhesion deficiency 1 (LAD1) is an inherited disorder of neutrophil function. Nonsense mutations in the affected CD18 (ITB2) gene have rarely been described. In other genes containing such mutations, treatments with aminoglycoside types of antibiotics (e.g., gentamicin) were reported to partially correct the premature protein termination, by induction of readthrough mechanism.

**Methodology/Principal Findings:**

Genetic analysis was performed on 2 LAD1 patients. Expression, functional and immunofluorescence assays of CD18 in the patients were used to determine the *in-vivo* and *in-vitro* effects of gentamicin-induced readthrough. A theoretical modeling of the corrected CD18 protein was developed to predict the protein function.

**Results:**

We found a novel premature termination codon, C562T (R188X), in exon 6 of the CD18 gene that caused a severe LAD1 phenotype in two unrelated Palestinian children. *In-vivo* studies on these patients' cells after gentamicin treatment showed abnormal adhesion and chemotactic functions, while *in-vitro* studies showed mislocalization of the corrected protein to the cytoplasm and not to the cell surface. A theoretical modeling of the corrected CD18 protein suggested that the replacement of the wild type arginine by gentamicin induced tryptophan at the position of the nonsense mutation, although enabled the expression of the entire CD18 protein, this was not sufficient to stabilize the CD18/11 heterodimer at the cell surface.

**Conclusion:**

A novel nonsense mutation in the CD18 gene causing a complete absence of CD18 protein and severe LAD1 clinical phenotype is reported. Both *in vivo* and *in vitro* treatments with gentamicin resulted in the expression of a corrected full-length dysfunctional or mislocalized CD18 protein. However, while the use of gentamicin increased the expression of CD18, it did not improve leukocyte adhesion and chemotaxis. Moreover, the integrity of the CD18/CD11 complex at the cell surface was impaired, due to abnormal CD18 protein and possibly lack of CD11a expression.

## Introduction

Leukocyte adhesion deficiency 1 (LAD1) is an inherited disorder of neutrophil function characterized by recurrent bacterial infections and impaired pus formation and wound healing [1]. The pathophysiology of LAD1 includes abnormalities of a wide variety of adhesion-dependent functions of hematopoietic cells due to deficiency of the beta-2 integrin (CD18, ITGB2) subunit [2]. Different types of mutations have been described in the CD18 gene [3]. These mutations interfere with the CD18/CD11 interaction and cause the lack of beta-2/alpha-L (CD18/CD11a), beta-2/alpha-M (CD18/CD11b), and beta-2/alpha-X (CD18/CD11c) expression. Nonsense mutations in the CD18 gene have rarely been described [4]. This type of mutation characteristically results in truncated or completely missing protein production and is associated with a severe disease phenotype. An aminoglycoside family of antibiotics (e.g., gentamicin) was recently reported to partially correct the effect of nonsense mutations by specifically recognizing ribosomes and by promoting a readthrough mechanism for the modulation of translation and miscoding [5]. The binding of aminoglycosides to ribosomes also enhances the ability of releasing factors, such as RF1 and RF2, to stabilize the nascent protein strand in the ribosome for further elongation [6]. Furthermore, the expression of various gene products associated with the translational machinery can be regulated by treating cells with aminoglycoside antibiotics [7]. Consequently, aminoglycoside antibiotics have been found to allow ribosomes to readthrough inappropriately inserted stop codon mutations in both human [8] and animal [9] models. The mechanism of translation termination is highly conserved among most organisms and is almost always signaled by an amber (UAG), ochre (UAA), or opal (UGA) termination codon [10]. By reducing the accuracy of translation, aminoglycosides increase the frequency of erroneous insertions at the nonsense codon and permit translation to continue to the end of the gene. Aminoglycoside antibiotics usually insert glutamine at nonsense UAG or UAA or tryptophan at nonsense UGA sites [11] albeit at extremely modest efficiencies of the affected genes. Indeed, patients suffering from different heritable diseases, such as cystic fibrosis, muscular dystrophies, hemophilia, lysosomal storage disorder or ataxia telangiectasia due to stop codon mutations experienced clinical and laboratory improvement after gentamicin treatment [12], [13], [14], [15], [16]. For example, expression of full-length CFTR protein at the apical cell membrane was observed in cystic fibrosis patients [17]. Moreover, suppression of stop mutations in the CFTR gene by parenteral gentamicin could be predicted *in-vitro* [18]. These clinical studies paved the way to the development of orally bioavailable small molecule modality that is designed to induce ribosomes to selectively read through some premature stop codons during mRNA translation, [19], however, raised some controversies regarding its application in other premature stop codons.

We describe here a novel premature termination codon in the CD18 gene causing severe LAD1 phenotype in two Palestinian children. We investigated the *in-vivo* and *in-vitro* effects of gentamicin-induced readthrough in the CD18 protein of these patients. We also show the effect of gentamicin treatment on the expression of CD11 molecules and their interaction with CD18 at the cell surface.

## Methods

### Patients

Two patients with a clinical phenotype suggestive of LAD1 and age-matched healthy control were studied. Parents provided signed informed consent to obtain blood from their children, to use tissues obtained from their children, to create cell lines and to test these samples for the effects of gentamicin treatment on the CD18 protein. Gentamicin was used purely for clinical purposes which were not related to this study. The Institutional Review Board (Sheba Medical Center, Tel Hashomer) approved human involvement, use of patient tissue and cell line creation. De novo lymphoclastiod cell lines were prepared. The consent and the IRB approval received extend to a lymphoblastoid cell line control that was previously approved by the institute IRB and published [20].

### Genetics

Genomic DNA was extracted from the studied patients' peripheral blood mononuclear cells (PBMCs). The CD18 gene was sequenced using the appropriate primers as previously described [21]. In addition, RNA was extracted (PerfectPure RNA Tissue kit,5′ prime, Germany) and cDNA was prepared (High capacity cDNA RT kit, Applied Biosystems,USA). The mutation region within CD18 transcripts obtained from both patients was PCR amplified and sequenced using the primers: Forward 5′- CCTCAACGAGATCACCGAGTC- 3′ and Reverse: 5′-GTTGCGCCAGCCGATTTCCTC- 3′.

### Cell lines

Epstein-Barr virus (EBV)-transformed lymphoblasts were prepared from lymphocytes obtained from each patient. These cell lines were maintained in RPMI 1640 supplemented with 10% fetal calf serum (FCS), pen-strep (100 mcg/ml) and 2 mM glutamine at 37°C in a humidified atmosphere of 5% CO_2_. The cells were grown in the presence or absence of various concentrations of gentamicin sulphate 50 mg/ml (Biological Industries Inc., Beit HaEmek, Israel), and were harvested at specified times.

### Neutrophil function

Neutrophil function was determined while both patients were receiving gentamicin treatment. The neutrophils were isolated (99% purity) from heparinized blood by dextran sedimentation, Histopaque gradient, and erythrocyte lysis, as described by Boyum [22]. They were re-suspended for chemotaxis studies in M199 medium (Biological Industries Inc., Beit HaEmek, Israel) (10^6^ cells/mL) and for adhesion in phosphate buffer saline (PBS) with 0.1% albumin and 0.2% glucose (5×10^6^ cells/mL). Chemotaxis was assessed in a 48-well chemotaxis chamber through a 3-mcm pore size filter [23] and induced by 1 mcM of the chemoattractant N-formyl-Met-Leu-Phe (fMLP) (Sigma-Aldrich, St. Louis, MO). Random migration was conducted in the presence of medium M199. Calculations of net chemotaxis were made by subtracting the random from the chemotactic migration (fMLP-stimulated neutrophils). All procedures were performed in quadruplicates. Cell adhesion to the gelatin surface was assessed as previously described [24]. Briefly, cells were labeled with 0.1% Calcein (MW 994.87; Molecular Probes-Eugene, Oregon) and incubated for 30 min at 37°C with either of the stimulates PMA (100 ng/ml) or fMLP (0.1 mcM) in a 24-well plate. HTAB lysis solution (0.1% Tween 20 [Sigma], 0.1% HTAB [Sigma], 0.2% BSA, 20 mM EDTA) was added. Fluorescence of the lysates was measured by a spectrofluorophotometer (Shimadzu, Tokyo, Japan) at an excitation wavelength of 485 nm and an emission of 535 nm.

### Flow cytometry analysis

Whole blood or transformed lymphoblasts from the patients and controls were collected and incubated with anti-CD18, anti-CD11a, anti-CD11b and anti-CD11c antibodies (Coulter Diagnostics). Red blood cells (RBCs) were lysed and white blood cells (WBCs) were washed with PBS. Lymphocytes, granulocytes and monocytes were gated. The cell surface expression of the various markers on these cells and the lymphoblasts was measured using flow cytometry (Epics V; Coulter Electronics, Hialeah, FL or Becton Dickinson CANTO II, BD Biosciences, NJ, USA, Diva software).

### Protein extraction and Western immunoblotting

EBV-transformed cells were treated with gentamicin (600, 1000 or 2000 mcg/ml) for 3 or 5 days. They were harvested and lysed with a lysis buffer containing 150 mM NaCl, 50 mM Hepes pH 7.4, 1 mM EDTA, 1 mM EGTA 1.5 mM MgCl 2, 1% triton-X, and 10% glycerol in the presence of a protease inhibitor cocktail (complete; Roche, Mannheim-Germany). Proteins were separated on 10% SDS-PAGE, transferred to a nitrocellulose membrane (Schleicher & Schuell, Germany) and probed with anti-CD18 antibody (abcam). ECL™ (Pharmacia Biotech, NJ, USA) chemiluminescent reagents were used for signal detection. Intensity of the band was analyzed using the Image EZ-Quant software package (EZ-Quante LTD., Israel).

### Immunofluorescent studies

EBV-transformed cells were treated with gentamicin (1000 mcg/ml) for 5 days, harvested and cytospan onto slides. They were then fixed with ice-cold methanol and ice-cold acetone, washed with Tris-buffered saline (TBS; 100 mM Tris-HCl pH 7.5, 150 mM NaCl) and blocked using 5% skim milk in TBS containing 0.1% Tween 20 (TBS-T). Mouse monoclonal anti-integrin B2 (CD18) antibody (Santa Cruz Biotechnology Inc., USA) was used to detect CD18 protein. The slides were mounted using immunofluore containing 4′,6-diamidino-2-phenylindolem (DAPI) for nuclear staining, and analyzed by optic grid fluorescent microscopy (Olympus).

### Structural bioinformatics

A 3D model of CD18 protein was built by homology modeling using Modeller [25]. A Cα-match server [26] was used to structurally align the model with ITB3 (CD61). Images were obtained using Chimera [27].

## Results

### Clinical findings

The two studied patients were both males of Palestinian descent. Patient #1 was 3 months old and Patient #2 was 12 months old at presentation. They belonged to two unrelated families and both were born to healthy consanguineous parents. Infantile fatalities at early age due to recurrent infections were known to have occurred in both families. The two patients had the typical clinical features of LAD1, including delayed umbilical cord separation, extremely high leukocyte counts, recurrent infections and abnormal wound healing. Their clinical details are summarized in Table 1. Patient 1 had inconsistent changes in leukocyte counts during and after treatment with gentamicin. Patient 2 displayed a consistent reduction in leukocyte counts during the treatment with gentamicin (from 41.3 to 21.9 cells per mm^3^). Yet both patients displayed a severe disease phenotype that resulted in the death of Patient #1 at **5** months of age due to severe pseudomonas infection before he could undergo bone marrow transplantation (BMT). Patient #2 underwent a BMT at age 15 months and currently is alive and well.

**Table 1:** Patient characteristics.

	Patient #1	Patient #2
Sex	Male	Male
Age at presentation, m	3	12
Age at umbilical cord separation	2.5	3
Family fatalities at early age	Positive	Positive
Type of infection	Deep and superficial abscesses, necrotic lesion (cervical)	Deep and superficial abscesses, necrotic lesions (wrist, thigh)
Wound healing	Abnormal	Abnormal
White blood count/neutrophils, mm^3^	54.8/34.6	41.3/24
Outcome	Death due to sepsis	Alive post bone marrow transplantation

### Genetics

**Figure 1:**
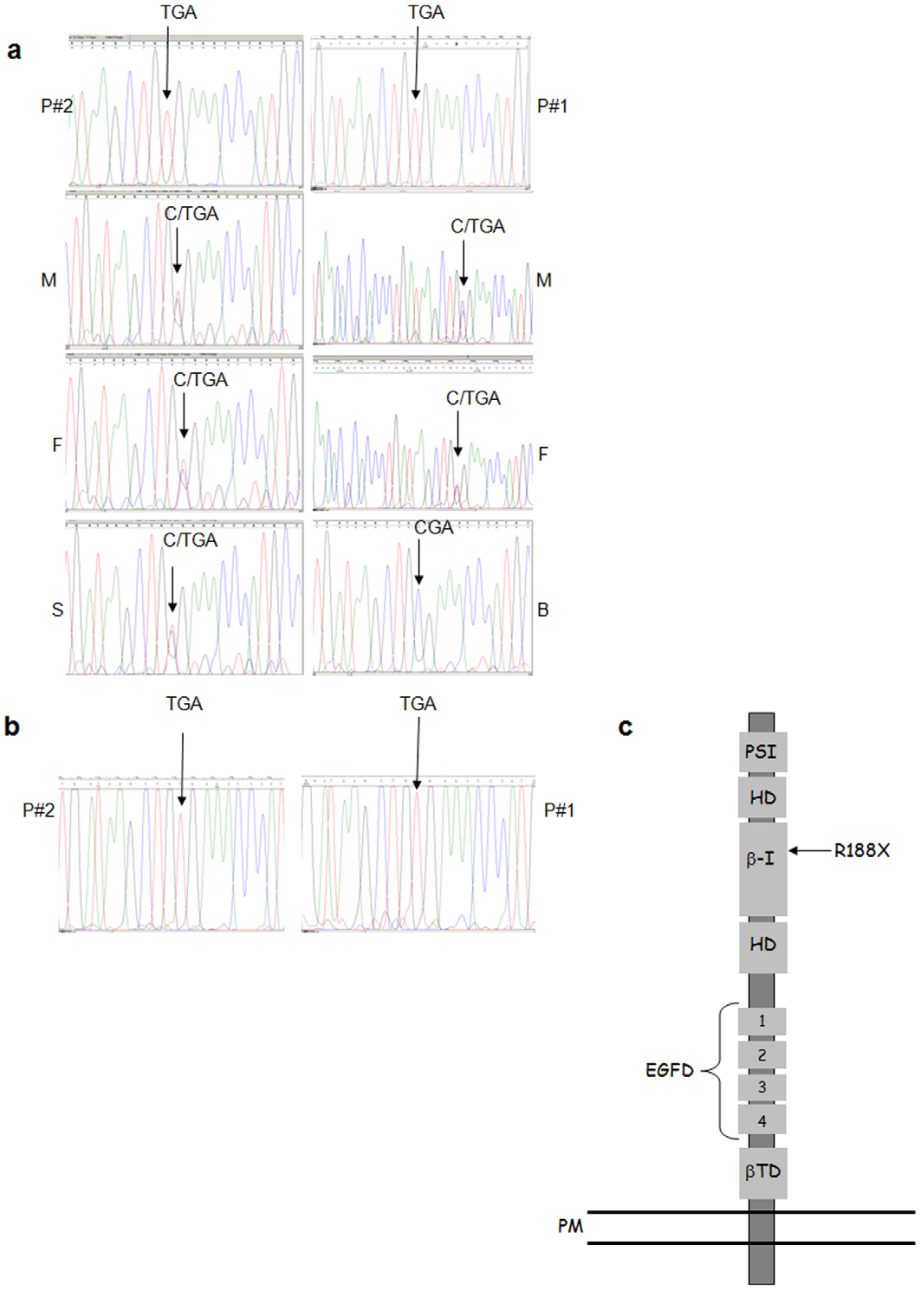
R188X nonsense mutation in ITGβ2/CD18. **a.** DNA sequences of the mutation region in exon 6 of CD18 gene in the two patients, their parents, one sister of patient #2 and one brother of patient #2. **b**. cDNA sequences of the mutation region in CD18 transcripts in the two patients **c.** Schematic structure of ITGβ2/CD18 protein, its domains and the position of the premature stop mutation. PSI indicates the plexin-semaphorin-integrin domain; HD, hybrid domain; EGFD, EGF-like domain; βTD, β-tail domain; PM, plasma membrane.

Sequence analysis of the patients' CD18 coding exons revealed a novel homozygous C562T nonsense mutation in exon 6 of the gene (Figure 1A). This mutation predicts a putative arginine to stop codon substitution (R188X) at the protein level (a codon change from CGA to TGA). This novel premature stop codon mutation resides within the highly conserved β-I domain of the various B-integrins including the ITB2/CD18 protein (Figure 1C). Therefore, we anticipated that such a mutation would yield either a 188 amino acid long truncated form of CD18 or the complete absence of this protein. Autosomal recessive inheritance of this mutation was confirmed by the heterozygosity of the mutated nucleotide in the asymptomatic parents and siblings of both families (Figure 1A).

### *In-vivo* induction of CD18 gene expression by gentamicin

#### Patient 1

**Figure 2:**
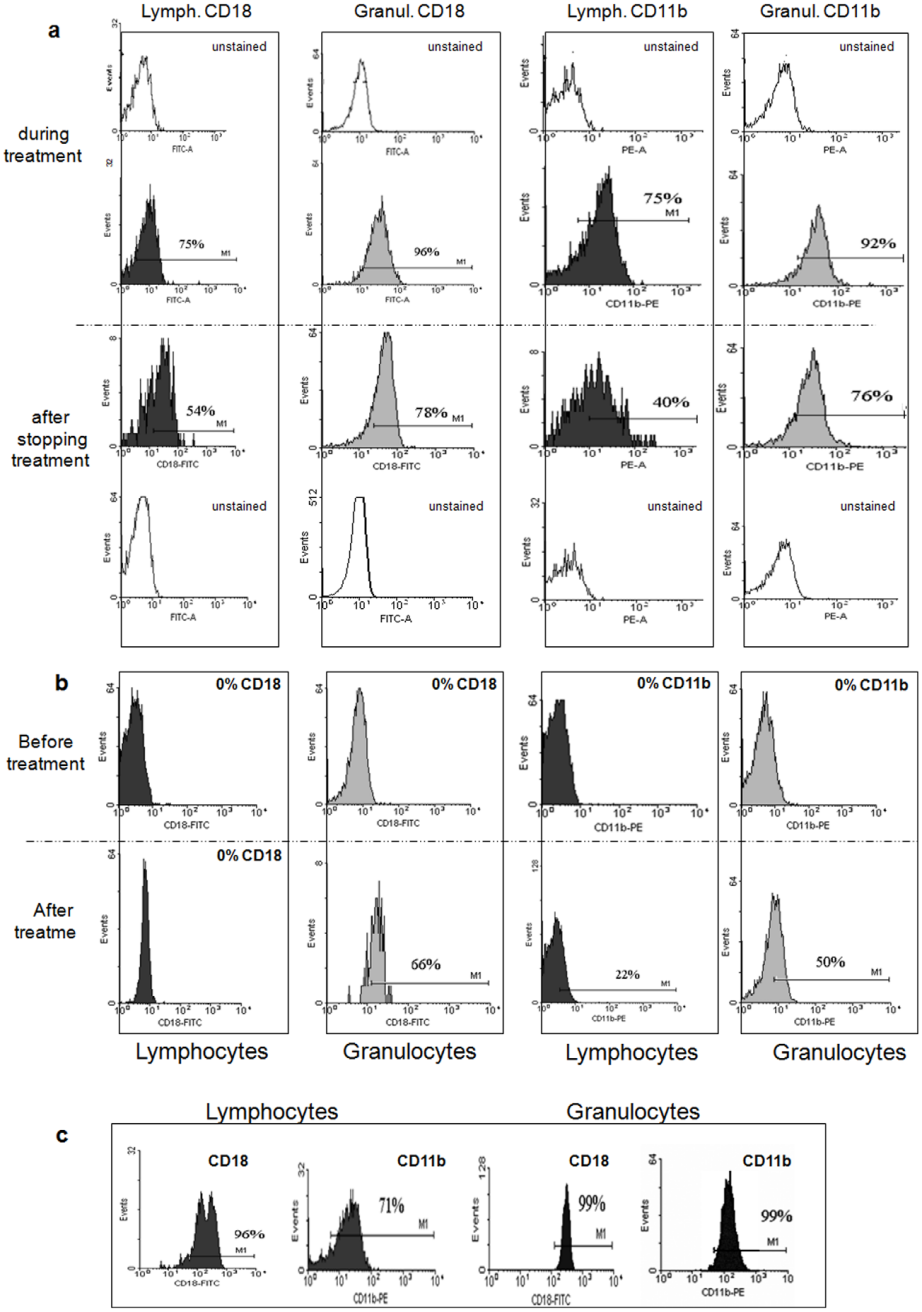
CD18 and CD11b cell surface expression upon gentamicin treatment *in vivo*. Whole blood samples from patient #1 (a), patient #2 (b) and healthy control (c) were incubated with anti-CD18 or anti-CD11b antibodies (Coulter Diagnostics). Lymphocytes (Lymph.) and granulocytes (Granul.) were gated and the expression of CD18 or CD11b on their cell surface was measured using flow cytometry (Epics V; Coulter Electronics, Hialeah, FL or Becton Dickinson CANTO II, BD Biosciences, NJ, USA, Diva software). Unstained cells obtained from patient #1 (a) were used as the control to set the M1 threshold. The percent of cells expressing the relevant cell surface marker in each case is indicated.

Upon admission, patient #1 was on gentamicin treatment for two weeks because of a skin infection. During this treatment, his CD11/CD18 workup showed detectable expression of the CD18 protein on total lymphocytes (75% of the cells), monocytes (93%) and granulocytes (96%), compared to a healthy control (94–99% of cells) (Figure 2 and Table 2). CD11b and CD11c were also detected while CD11a was undetected (Table 2). One week later, gentamicin was stopped due to renal toxicity and acute renal failure. A repeated immune workup that was performed one week after stopping gentamicin treatment revealed significant reduction in the expression of the CD18 protein on total lymphocytes (54%), monocytes (35%) and granulocytes (78%) (Figure 2 and Table 2). In addition, reduced expression of CD11b and CD11c on different cell types was detected, while CD11a remained undetectable (Table 2).

**Table 2:** Expression of CD11/18 in patients 1 and 2 treated with or without gentamicin.

	Cell	CD18		CD11a		CD11b		CD11c	
GentamicinTreatment		**-**	**+**	**-**	**+**	**-**	**+**	**-**	**+**
Patient #1	Lymph	54 (19)	75 (8)	0	0	40 (12)	75 (16)	0	25
	Mono	35	93	0	0	40	87	52	48
	Gran	78 (40)	96 (31)	0	0	76	92	72	71
Patient #2	Lymph	0	0	0	0	0	22	0	0
	Mono	0	0	0	0	0	54	32	55
	Gran	0 (7)	66 (16)	0	0	0 (5)	50 (7)	9	82
Control	Lymph	96		76		71		8	
	Mono	94		94		95		96	
	Gran	99		85		99		60	

Results are given in percents. When weak expression and/or low number of events were detected, MFI (mean fluorescence intensity) was measured as well, and results are presented in brackets.

Lymph indicates lymphocytes; Mono, monocytes; Gran, granulocytes.

#### Patient 2

On presentation, patient #2 had undetectable expression of CD18/11 heterodimers on lymphocytes, expression of CD11c (32% of cells) on monocytes and expression of CD11c (9%) on granulocytes. CD11a was not detected on any of the examined cells (Table 2). The immune workup was repeated after two weeks of gentamicin treatment that was empirically prescribed for an acute febrile episode. Surprisingly, the expression of CD18 was significantly increased on the granulocytes (66%). In addition, the expression of CD11b and CD11c on different cell types had increased while CD11a remained undetectable (Figure 2 and Table 2). In order to verify that the elevation in CD18 expression was not due to a reversion in the mutated CD18 transcript but rather due to post-translation change in the protein, RNA was extracted from lymphocytes of both patients during gentamicin treatment, cDNAs were prepared and CD18 transcripts were sequenced. In both samples the C562T (R188X) germline mutation was found, suggesting post-translation gentamicin-induced correction as expected (Figure 1B).

### *In-vitro* induction of CD18 protein expression by gentamicin

**Figure 3:**
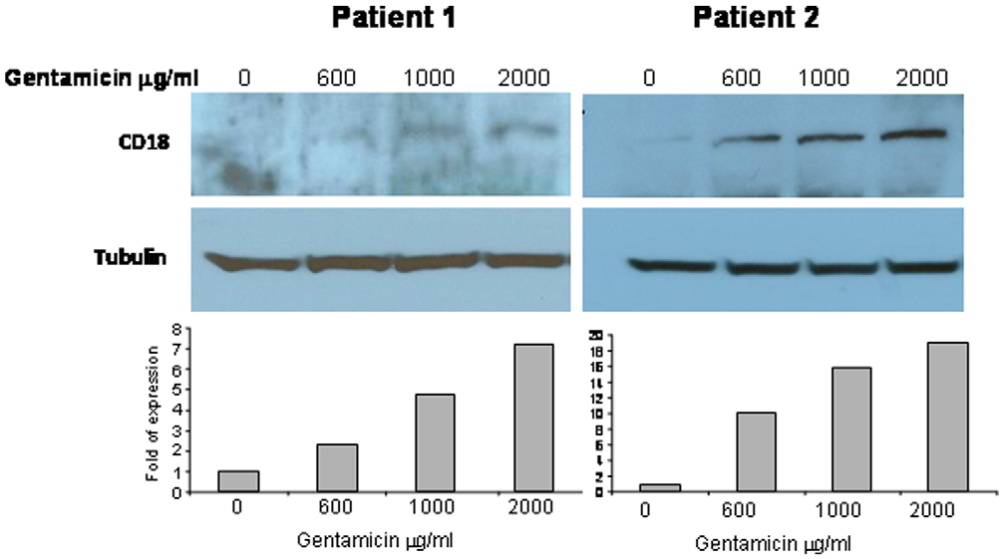
*In vitro* induction of CD18 protein expression by gentamicin treatment. The two patients' Epstein-Barr virus-transformed cells were treated with increasing concentrations of gentamicin for 3 days. Proteins were extracted from whole cell lysates for western blot analysis (upper panels) using anti-CD18 antibodies (abcam) and anti-tubulin antibody (Sigma) for equal loading control. Intensities of the CD18 bands were calculated using Image EZ-Quant software package (EZ-Quante LTD., Israel), normalized to the untreated samples (lower panels).

EBV-transformed lymphoblasts from both patients were treated for 3–5 days with incremental doses of gentamicin (600–2000 mcg/ml). The cell surface expression of the CD11/18 heterodimers was determined by FACS analysis. The expression of CD18 and CD11 proteins in both cell lines was undetectable or very low before gentamicin treatment and increased slightly with gentamicin treatment (Table 3). There was a similar trend for CD18/11 expression at both 3 and 5 days of gentamicin treatment. The CD18 level had increased to a maximal expression of 2% after 3 days of treatment with 600 mcg/ml gentamicin. Interestingly, unlike its undetectable *in-vivo* expression in the patients' cells, a slight expression of CD11a was detected after gentamicin treatment (Table 3). The protein expression level of CD18 was detected by western blot (Figure 3): expression of the CD18 protein (95 KD band) had increased after 3 days following gentamicin treatment. This effect was dose-dependent, reaching a maximal effect after 3 days of 2000 mcg/ml gentamicin treatment. These results showed that the slight elevation of CD18 expression as detected by FACS analysis upon gentamicin treatment did not correlate with the elevation of the protein level as detected by western blot. This finding suggested that although gentamicin induced high expression of CD18 protein, only part of the protein was expressed on the cell surface as detected by FACS. Immunolocalization studies of the CD18 protein during treatment with gentamicin were performed to check this possibility.

**Table 3:** *In-vitro* induction of CD18.

	Treatment	CD18	CD11a	CD11b	CD11c
Patient #1	-	0	0	0.8	0.4
	600	0.6	0	1.1	0.5
	1000	1	0	0.3	0.2
	2000	0.5	0	0.8	0.2
Patient #2	-	0	0	33	23
	600	2	1.7	34	11
	1000	0	0.9	14	6
	2000	0	1.6	8	6
Control	-	81	88	22	30

### Immunolocalization of CD18

**Figure 4:**
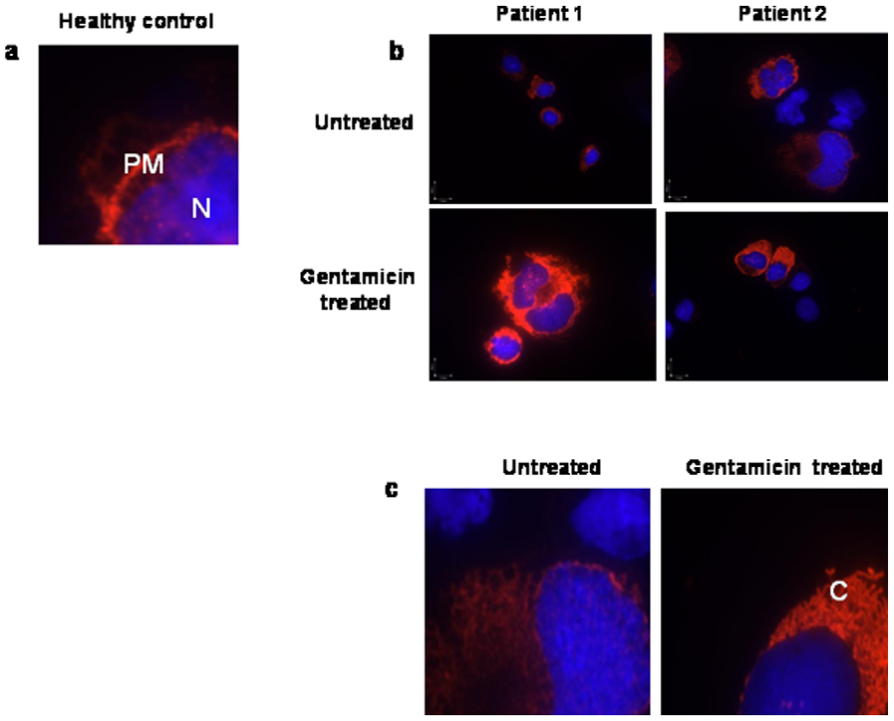
Immuno-localization of CD18 upon treatment with gentamicin. The two patients' Epstein-Barr virus-transformed cells were treated with gentamicin (1000 µg/ml) for 5 days. Cells were cytospun, fixed and immunostained with mouse monoclonal anti integrin β2 (CD18) antibody (Santa Cruz Biotechnology Inc., USA). Slides were mounted using immunofluorescence and DAPI for nuclear staining and analyzed by optic grid fluorescence microscopy (Olympus). **a.** 1000-fold magnification of healthy control's Epstein-Barr virus-transformed lymphocytes **b.** 600-fold magnification of gentamicin untreated (upper panel) and treated (lower panel) lymphoblastoids derived from patients 1 and 2. **c.** 1000-fold magnification of gentamicin untreated (left panel) and treated (right panel) lymphoblastoids derived from patient 1. N indicates nucleus; PM, plasma membrane; C, cytoplasm.

To test our hypothesis that gentamicin-induced readthrough caused intact protein formation but not the appropriate localization on the cell surface, lymphoblast cells derived from the two patients were treated with gentamicin and harvested for immunofluorescence staining. Their CD18 immuno-localization was compared to that of untreated lymphoblasts derived from healthy control (Figure 4). After undergoing gentamicin treatment, cells from both patients showed cytoplasmic staining but not cell membrane staining of the CD18 protein. In contrast, cells from healthy control showed clear staining of CD18 at the cell membrane (Figure 4).

### Chemotaxis and cell adhesion

Upon neutrophil stimulation with fMLP and PMA, the adhesion index in both patients was unchanged and remained 1. In contrast, two healthy controls exhibited significantly increased adhesion index (values of 3.3–7.2 and 9.6–9.8 following fMLP and PMA stimulations, respectively). An increased adhesion index was observed also in the patients' mothers upon neutrophil stimulation (2.3–3.3 and 4.9–5.1 for fMLP and PMA stimulation, respectively. Both mothers were heterozygote carriers of the CD18 nonsense mutation). Thus, the net chemotaxis (fMLP-stimulated minus unstimulated cells) of both patients was significantly reduced (4 and 19 cells/field) compared to the healthy controls (42–57 cells/field) and to their mothers (41 and 75 cells/field). These findings indicate a significant neutrophil dysfunction.

### Theoretical modeling of the CD18 protein

**Figure 5:**
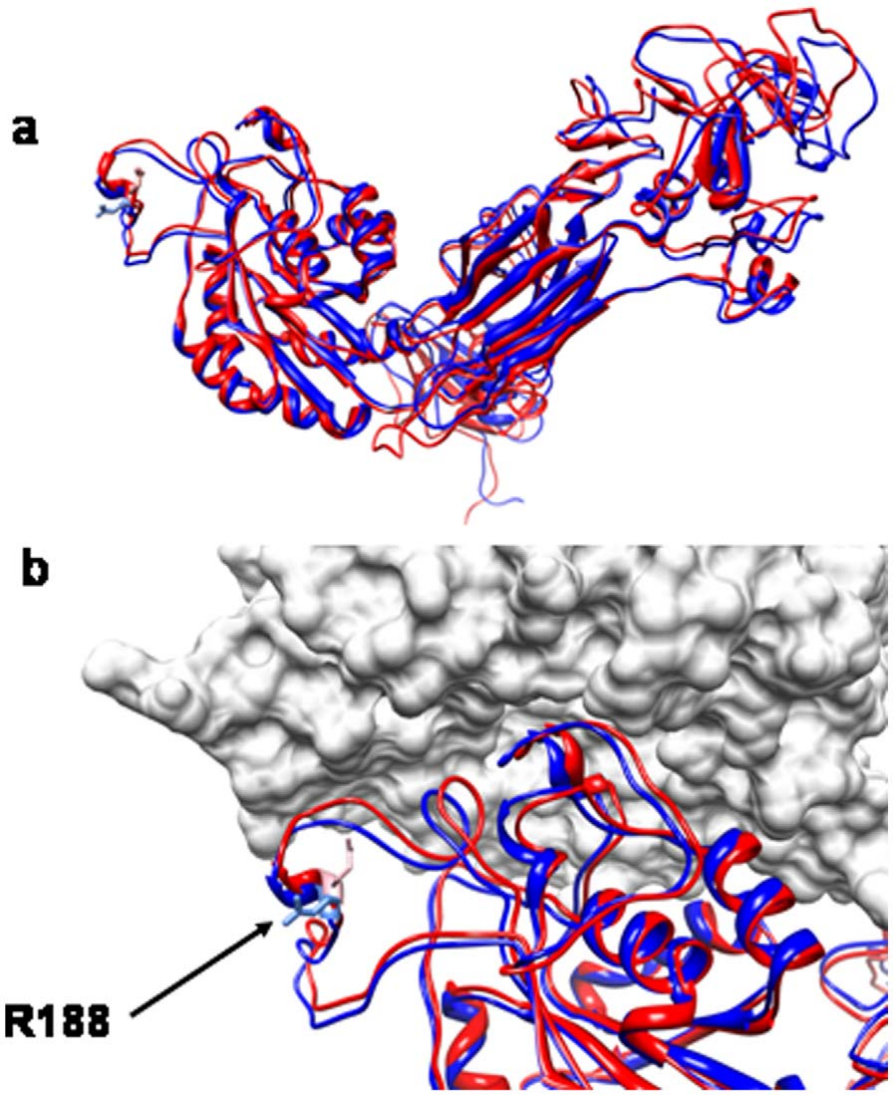
Theoretical model of human CD18. **A.** A 3-D model of CD18 (blue) was built by homology modeling using ITGβ3 (CD61) structure as a template (red, PDB code 3ije). **B.** Superposition of the model with αvβ3 (CD51/CD61) complex (PDB code 1l5g). The β-I domains (CD18 in the model [blue]), CD61 in the template [red]) are given in cartoon representation with the arrow pointing to the position of the R188 residue, mutated in the LAD1 patients. The ITGαv subunit (CD51) is displayed in a surface representation.

In order to better understand why the corrected gentamicin-induced readthrough CD18 was mislocalized, we computerized a homologous model of CD18 containing the wild-type arginine residue or the replaced putative tryptophan residue (Figure 5). Upon gentamicin treatment, it is assumed that this residue was inserted during translation instead of the stop codon at amino acid position 188. The model suggests that tryptophan is located in a relatively exposed and flexible loop in CD18 which is not conserved between the integrin B-subunits. Superimposing the model on top of an experimentally available B-subunit in complex (αvβ3 complex) allowed us to predict that this residue is in close proximity to the α-β interface, assuming that the interface is similar to that which is between CD18 and its α-subunit partners.

## Discussion

Nonsense mutations have rarely been described in the CD18 gene [4]. This type of mutation was reported to correlate with a severe clinical phenotype in other primary immunodeficiencies, such as Wiskott-Aldrich syndrome, [28], as well as in non-immunodeficiency inherited diseases [29]. Herein, we describe a novel R188X nonsense mutation in the CD18 gene in two children of Palestinian descent with a possible common founder probound. A normal heterozygote carrier for this mutation has been reported [30]. Both patients described by us, had significantly impaired wound healing in addition to the other typical LAD1 clinical characteristics. Interestingly, this severe clinical manifestation resembles that of CD18 null mice [31]. The latter was shown to suffer from spontaneous skin ulceration and chronic dermatitis with extensive facial and submandibular erosions, in addition to the typical LAD1 clinical findings. It can be concluded that the severe and identical clinical phenotype in both humans and mice is attributable to the complete absence of the CD18 protein. Therefore, restoring this protein and its function in LAD1 patients is of great importance.

To date, the only curative treatment in such severe cases of LAD1 is allogeneic BMT [32], [33]. However, alternative treatments are often required for patients who are waiting to undergo a BMT or when the procedure is not available. One of these alternatives may be the correction of the nonsense mutated dysfunctional CD18 protein by the gentamicin-induced readthrough mechanism. The identification of a clinically feasible method to suppress premature stop mutations within the CD18 gene might be of considerable benefit to patients with LAD1 as well as to those with other diseases caused by stop mutations. We sought to determine whether this mechanism is applicable to the specific nonsense mutation carried in the CD18 gene of two patients while they were awaiting BMT. Before treatment, the reported mutation resulted in undetectable CD18 protein levels in both patients' cells, either because the putative truncated protein was completely missing or it was misfolded. Our results suggest that gentamicin treatment induced readthrough of the mutated gene, yielding a corrected full-length CD18. The fact that sequencing of both patients cDNA revealed the germline mutation suggests a post-translation gentamicin-induced correction and not a reversion of the mutated CD18 gene. This treatment did not, however, improve the clinical manifestation of the condition or the leukocyte adhesion and chemotaxis. Indeed, the gentamicin-induced corrected full-length protein was either dysfunctional or mislocalized.

The novel nonsense type of mutation that we found enabled us to test the proof of principle that interventions designed to read through premature stop mutations may at least partially reverse a clinical phenotype. Our study is important in light of the controversies regarding the effect of gentamicin-induced readthrough that exists even in the same diseases [34], [35]. In general, targets with the UGA stop codon, such as that displayed in our patients, showed a higher translational readthrough than those with other stop codon changes [10]. We showed increased production of the entire CD18 protein (western blot analysis) in the lymphoblasts after treatment with gentamicin. Since stop codons in various genes show a broad spectrum of readthrough efficiency in response to gentamicin, the extent of increase in CD18 expression that followed this treatment was surprisingly high. For example, patients with muscular dystrophies [35] and cystic fibrosis [18] were shown to have readthrough levels of the affected gene ranging from only 0.05% to 2.65%. Despite the significant elevation in the expression of the corrected CD18, only a slight change in the cell surface expression of the heterodimer CD11/18 was demonstrated in lymphoblasts after gentamicin treatment (up to 2% CD18), as demonstrated both by FACS analysis and immunofluorescent studies of these cells. This non-functional expression of the heterodimer may explain the failure or the relative difficulty to obtain significant changes in leukocyte functions.

CD18 expression is required for the normal expression of the CD11 components of the heterodimers [36], yet their expression does not confirm function [37]. Differences in CD18/CD11 expression in LAD1 patients due to the different homeostasis of their leukocytes have never been addressed before. By testing the effect of gentamicin on the CD18 level, we could also determine the effect of the corrected CD18 on the proper cell surface expression of the different CD11 molecules and the integrity of the entire CD18/11 complex. Interestingly, while CD11b and CD11c were positively affected by the increased level of CD18, CD11a remained undetectable by FACS analysis. Therefore, we can speculate that a gentamicin-induced readthrough has an effect on CD18 expression which allows its ability to associate with the CD11b and CD11c subunits but not with the CD11a subunit at the cell surface. We hypothesize that the inability of the corrected CD18 to bind CD11a is due to the gentamicin induced replacement of tryptophan residue instead of the wild type arginine at amino acid position 188 of CD18 protein, as suggested also by our computerized modeling analysis of the predicted heterodimer. While the truncated CD18 protein that resulted from the premature stop codon was either degraded or misfolded and therefore dysfunctional, it is likely that the corrected CD18 was not properly folded because the tryptophan replacement is not located in the protein core. Since there is no available structure to depict the CD18/CD11 complex, our proposed model and its orientation with respect to the CD51/CD61 resolved complex suggests that the tryptophan is located close to the interface and might interact with or sterically disturb any interaction with its alpha chain partners, such as CD11a. In any event, the charge difference (i.e., aromatic tryptophan replacing positively charged arginine) might affect binding, even in longer range interactions.

Since we could not significantly affect the neutrophil function despite elevating the expression of CD18, CD11b and CD11c, we can also speculate that CD11a is a crucial factor for the CD18/11 adhesion-dependent functions of hematopoietic cells [38]. Indeed, the involvement of CD18/CD11a heterodimer in the adhesion of cytotoxic T cells to their target cells, including the delivery of a distinct signal essential for directing released granules to antigen-bearing target cells to mediate their destruction, is unique [39]. An alternative explanation for the dysfunctional cells is that expression of the other CD18/11 components were not enough, thus a higher level of readthrough would be required in order to restore appropriate localization of the CD18 protein and full functional activity. This speculation can provide a rationale for the development of future therapeutic modalities aiming to maximize the readthrough potential or to improve the function of the corrected protein at the cell surface. For example, Nudelman et al. [40] recently described a novel NB54 aminoglycoside with reduced toxicity and enhanced suppression of disease-causing premature stop mutations in cells that originated from various hereditary diseases which improves both expression and function of the corrected protein.

In summary, we presented a novel premature termination codon mutation in the CD18 gene of two Palestinian children with severe LAD1 phenotype. This mutation enabled us to test the proof of concept designed to readthrough premature stop mutations. We showed that a corrected full-length CD18 is produced as a result of gentamicin treatment both *in vivo* and *in vitro,* although the protein is either dysfunctional or mislocalized. In addition, we found that the integrity of the CD18/CD11 complex was impaired, possibly due to lack of CD11a expression. Our results should encourage the search for more effective aminoglycoside readthrough compounds to treat LAD1 and other potential genetic disorders caused by nonsense mutations.

## References

[1] Dinauer MC (2007). Disorders of neutrophil function: an overview.. Methods Mol Biol.

[2] Bunting M, Harris ES, McIntyre TM, Prescott SM, Zimmerman GA (2002). Leukocyte adhesion deficiency syndromes: adhesion and tethering defects involving beta 2 integrins and selectin ligands.. Curr Opin Hematol.

[3] Wardlaw AJ, Hibbs ML, Stacker SA, Springer TA (1990). Distinct mutations in two patients with leukocyte adhesion deficiency and their functional correlates.. J Exp Med.

[4] Fiorini M, Piovani G, Schumacher RF, Magri C, Bertini V (2009). ITGB2 mutation combined with deleted ring 21 chromosome in a child with leukocyte adhesion deficiency.. J Allergy Clin Immunol.

[5] Xi B, Guan F, Lawrence DS (2004). Enhanced production of functional proteins from defective genes.. J Am Chem Soc.

[6] Karimi R, Pavlov MY, Buckingham RH, Ehrenberg M (1999). Novel roles for classical factors at the interface between translation termination and initiation.. Mol Cell.

[7] Murphy GJ, Mostoslavsky G, Kotton DN, Mulligan RC (2006). Exogenous control of mammalian gene expression via modulation of translational termination.. Nat Med.

[8] Hermann T (2007). Aminoglycoside antibiotics: old drugs and new therapeutic approaches.. Cell Mol Life Sci.

[9] Guerin K, Gregory-Evans CY, Hodges MD, Moosajee M, Mackay DS (2008). Systemic aminoglycoside treatment in rodent models of retinitis pigmentosa.. Exp Eye Res.

[10] Martin R, Mogg AE, Heywood LA, Nitschke L, Burke JF (1989). Aminoglycoside suppression at UAG, UAA and UGA codons in Escherichia coli and human tissue culture cells.. Mol Gen Genet.

[11] Nilsson M, Rydén-Aulin M (2003). Glutamine is incorporated at the nonsense codons UAG and UAA in a suppressor-free Escherichia coli strain.. Biochim Biophys Acta.

[12] Rowe SM, Varga K, Rab A, Bebok Z, Byram K (2007). Restoration of W1282X CFTR activity by enhanced expression.. Am J Respir Cell Mol Biol.

[13] Wagner KR, Hamed S, Hadley DW, Gropman AL, Burstein AH (2001). Gentamicin treatment of Duchenne and Becker muscular dystrophy due to nonsense mutations.. Ann Neurol.

[14] Pinotti M, Rizzotto L, Pinton P, Ferraresi P, Chuansumrit A (2006). Intracellular readthrough of nonsense mutations by aminoglycosides in coagulation factor VII.. J Thromb Haemost.

[15] Brooks DA, Muller VJ, Hopwood JJ (2006). Stop-codon read-through for patients affected by a lysosomal storage disorder.. Trends Mol Med.

[16] Lai CH, Chun HH, Nahas SA, Mitui M, Gamo KM (2004). Correction of ATM gene function by aminoglycoside-induced read-through of premature termination codons.. Proc Natl Acad Sci USA.

[17] Wilschanski M, Yahav Y, Yaacov Y, Blau H, Bentur L (2003). Gentamicin-induced correction of CFTR function in patients with cystic fibrosis and CFTR stop mutations.. N Engl J Med.

[18] Sermet-Gaudelus I, Renouil M, Fajac A, Bidou L, Parbaille B (2007). In vitro prediction of stop-codon suppression by intravenous gentamicin in patients with cystic fibrosis: a pilot study.. BMC Med.

[19] Kerem E, Hirawat S, Armoni S, Yaakov Y, Shoseyov D (2008). Effectiveness of PTC124 treatment of cystic fibrosis caused by nonsense mutations: a prospective phase II trial.. Lancet.

[20] Margalit O, Amram H, Amariglio N, Simon AJ, Shaklai S (2006). BCL6 is regulated by p53 through a response element frequently disrupted in B-cell non-Hodgkin lymphoma.. Blood.

[21] Roos D, Meischl C, de Boer M, Simsek S, Weening RS (2002). Genetic analysis of patients with leukocyte adhesion deficiency: genomic sequencing reveals otherwise undetectable mutations.. Exp Hematol.

[22] Böyum A (1968). Isolation of mononuclear cells and granulocytes from human blood. Isolation of monuclear cells by one centrifugation, and of granulocytes by combining centrifugation and sedimentation at 1 g.. Scand J Clin Lab Invest.

[23] Falk W, Goodwin RH, Leonard EJ (1980). A 48-well micro chemotaxis assembly for rapid and accurate measurement of leukocyte migration.. J Immunol Methods.

[24] Petersen TK, Bysted BV, Jensen AL (1999). Determination of the adhesive capability of canine polymorphonuclear neutrophil granulocytes using a fluorometric microtiter plate cellular adhesion assay.. Vet Immunol Immunopathol.

[25] Sali A, Blundell TL (1993). Comparative protein modelling by satisfaction of spatial restraints.. J Mol Biol.

[26] Bachar O, Fischer D, Nussinov R, Wolfson HJ (1993). A computer vision based technique for 3-D sequence-independent structural comparison of proteins.. Protein Eng.

[27] Pettersen EF, Goddard TD, Huang CC, Couch GS, Greenblatt DM (2004). UCSF Chimera—a visualization system for exploratory research and analysis.. J Comput Chem.

[28] Jin Y, Mazza C, Christie JR, Giliani S, Fiorini M (2004). Mutations of the Wiskott-Aldrich Syndrome Protein (WASP): hotspots, effect on transcription, and translation and phenotype/genotype correlation.. Blood.

[29] Schorry EK, Keddache M, Lanphear N, Rubinstein JH, Srodulski S (2008). Genotype-phenotype correlations in Rubinstein-Taybi syndrome.. Am J Med Genet A.

[30] Lorusso F, Kong D, Jalil AK, Sylvestre C, Tan SL (2006). Preimplantation genetic diagnosis of leukocyte adhesion deficiency type I.. Fertil Steril.

[31] Scharffetter-Kochanek K, Lu H, Norman K, van Nood N, Munoz F (1998). Spontaneous skin ulceration and defective T cell function in CD18 null mice.. J Exp Med.

[32] Thomas C, Le Deist F, Cavazzana-Calvo M, Benkerrou M, Haddad E (1995). Results of allogeneic bone marrow transplantation in patients with leukocyte adhesion deficiency.. Blood.

[33] Linde L, Kerem B (2008). Introducing sense into nonsense in treatments of human genetic diseases.. Trends Genet.

[34] Bidou L, Hatin I, Perez N, Allamand V, Panthier JJ (2004). Premature stop codons involved in muscular dystrophies show a broad spectrum of readthrough efficiencies in response to gentamicin treatment.. Gene Ther.

[35] Clancy JP, Rowe SM, Bebok Z, Aitken ML, Gibson R (2007). No detectable improvements in cystic fibrosis transmembrane conductance regulator by nasal aminoglycosides in patients with cystic fibrosis with stop mutations.. Am J Respir Cell Mol Biol.

[36] Hynes, RO (1992). Integrins: versatility, modulation and signaling in cell adhesion.. Cell.

[37] Hogg N, Stewart MP, Scarth SL, Newton R, Shaw JM (1999). A novel leukocyte adhesion deficiency caused by expressed but nonfunctional beta2 integrins Mac-1 and LFA-1.. J Clin Invest.

[38] Larson, RS, Corbi AL, Berman L, Springer T (1989). Primary structure of the leukocyte function-associated molecule-1 alpha subunit: an integrin with an embedded domain defining a protein superfamily.. J Cell Biol.

[39] Anikeeva N, Somersalo K, Sims TN, Thomas VK, Dustin ML (2005). Distinct role of lymphocyte function-associated antigen-1 in mediating effective cytolytic activity by cytotoxic T lymphocytes.. Proc Nat Acad Sci.

[40] Nudelman I, Rebibo-Sabbah A, Cherniavsky M, Belakhov V, Hainrichson M (2009). Development of novel aminoglycoside (NB54) with reduced toxicity and enhanced suppression of disease-causing premature stop mutations.. J Med Chem.

